# Multimodality Imaging in Carotid Web

**DOI:** 10.3389/fneur.2019.00220

**Published:** 2019-03-12

**Authors:** Thomas P. Madaelil, Jonathan A. Grossberg, Raul G. Nogueira, Aaron Anderson, Clara Barreira, Michael Frankel, Diogo C. Haussen

**Affiliations:** Departments of Neurology and Neurosurgery, Marcus Stroke and Neuroscience Center, Grady Memorial Hospital, Emory University School of Medicine, Atlanta, GA, United States

**Keywords:** fibromuscular dysplasia, carotid web, stroke, diagnostic imaging, neuroradiology

## Abstract

**Purpose:** Carotid web (CaW) is an underrecognized cause of cryptogenic stroke in young patients. The optimal imaging for CaW is unknown. We aim to evaluate the diagnostic accuracy of diverse imaging modalities for the diagnosis of CaW.

**Methods:** Retrospective analysis of institutional neurovascular database was performed to identify patients with multimodal (CT angiogram–CTA, digital subtraction angiogram–DSA, and/or ultrasound–US) imaging diagnosis of CaW or atherosclerosis. Baseline clinical demographics were recorded. Blinded image analysis was performed for each imaging modality by separate readers. Discrepancies were settled by consensus. Two-sided Cohen's Kappa (κ) coefficient was used to evaluate the inter-rater agreement for the etiological diagnosis between imaging modalities.

**Results:** Thirty patients/60 carotids were evaluated by CTA and 55 carotids were included. Patients with symptomatic CaW (*n* = 20), compared to individuals with atherosclerosis (*n* = 10), were younger (49 ± 9 vs. 60 ± 8 years; *p* < 0.01), more commonly female (75% vs. 30%; *p* = 0.01), and less frequently presented vascular risk factors: Hypertension (40% vs. 100%; *p* < 0.01), hyperlipidemia (0% vs. 50%; *p* < 0.01), diabetes (10% vs. 40%; *p* = 0.05), and smoking (5% vs. 70%; *p* < 0.01). High inter-rater correlation strength existed for CTA (*n* = 55; κ = 0.88; *p* < 0.0001) and DSA (*n* = 28; κ = 0.86, *p* < 0.0001) readers for lesion diagnosis while US inter-rater agreement was lower (κ = 0.553; *p* = 0.001). Across modalities CTA and DSA shared very high strength of agreement (κ = 0.92; *p* < 0.0001), compared to a less pronounced agreement between US and CTA (κ = 0.553; *p* = 0.001). The strength of correlation between DSA-CTA was significantly more robust as compared to US-CTA (Z = 3.58; *p* = 0.0003).

**Conclusion:** CTA and DSA demonstrated comparable and superior performance as compared to US in the diagnosis of CaW.

## Introduction

Carotid web (CaW) is a variant of intimal fibromuscular dysplasia and an under-recognized cause of recurrent ischemic strokes in young patients (1). The lesion is characterized by an endoluminal projectile fibrotic mass that generates flow stagnation and, consequently, thrombo-embolic events (2, 3). Due to the reported high-rates of recurrent stroke despite antiplatelet use, dual antithrombotics, anticoagulation and revascularization have been proposed (1). Therefore, appropriate diagnosis is imperative.

Although first described on catheter angiography (4), non-invasive modalities such as carotid ultrasound (US) (5) and computed tomographic angiography (CTA) (3) can be diagnostic for CaW. In the setting of transient ischemic attacks or stroke, both CTA and US are routinely performed for non-invasive evaluation; however, the relative accuracy for the diagnosis of CaW across these modalities has never been investigated. The objective of the present study is to evaluate the diagnostic performance of different imaging techniques for the detection of CaW.

## Methods

Using an institutional neurovascular database, we retrospectively identified consecutive patients diagnosed with CaW and internal carotid artery atherosclerosis. Patient inclusion criteria comprised multimodality imaging comprising neck CTA at baseline plus either Digital Subtraction Angiography (DSA) and/or carotid US. This study was approved by the Institutional Review Board.

### Patient Selection

All consecutive patients with cryptogenic strokes or transient ischemic attack (TIA) attributed to a symptomatic CaW were included spanning August 2014 to June 2017. CaW were diagnosed in cryptogenic ischemic strokes (undetermined stroke etiology—negative investigation subtype) by TOAST criteria (Trial of Org 10172 in Acute Stroke Treatment classification) (6). Minimum workup included non-contrast brain CT and MRI Brain, head and neck CT angiogram (CTA), coagulation tests, inpatient telemetry, electrocardiogram, plus trans-thoracic and/or trans-esophageal echocardiography in accordance with AHA Stroke Council and Stroke Association guidelines (7). Patients with an atherosclerotic symptomatic internal carotid artery were randomly selected from an endovascular database within the same study period in a proportion of 2:1 CaW:atherosclerosis. A symptomatic lesion was defined by a CaW or carotid artery bulb atherosclerotic lesion attributed as the cause of a TIA or stroke. Baseline clinical demographic information was recorded.

### Evaluation of Lesion Etiology

CTA has been considered the modality of choice to diagnose CaW and atherosclerosis since its widely available, permits detailed non-invasive anatomic imaging, multi-plane reconstruction, short acquisition time, and reliable vessel wall imaging (2, 3, 8–11). CTA was therefore used in the present study to establish the etiological diagnosis and then directly compared to the other imaging modalities (CTA vs. US and CTA vs. DSA). Carotids that could not be evaluated by CTA, due to cervical internal carotid occlusion, artifact, or superimposed thrombus without follow-up imaging (to evaluate for underlying etiology) were excluded. Patients with diagnosis of stroke caused by CaW identified as having superimposed atherosclerostic calcific changes were excluded. Each patient's symptomatic carotid artery as well as the contralateral asymptomatic carotid were evaluated for the presence of (1) CaW, (2) atherosclerotic plaque, or (3) normal findings by each of the available imaging methods.

### CTA

Two blinded readers (neuroradiologist and neurointerventionist #1) independently evaluated CTAs obtained on a GE Revolution 256 slice CT scanner or GE Lightspeed 64 slice CT scanner (GE Healthcare; Chicago, IL) on iSite PACS (Phillips North America; Andover, MA). Individual carotid arteries were evaluated independently, without knowledge of clinical information or appearance of the contralateral carotid. Discrepant opinions were settled by consensus. All carotids were evaluated in axial (thin cuts), coronal, sagittal and oblique projections. A CaW was defined as a thin shelf-like intraluminal filling defect along the posterior wall of the carotid bulb just beyond the carotid bifurcation. Atherosclerotic lesions were defined by the presence of a plaque leading to vessel wall thickness >2.2 mm (12) involving the distal common carotid or proximal internal carotid in a non-focally projectile morphology with or without the presence of superimposed calcification. The degree of stenosis was recorded based on NASCET criteria (mild: 1–29%, low moderate: 30–50%, high-moderate: 50–69%, severe: 70–99%) (13, 14). In addition, vessel wall calcification was identified as any hyperdense focus with relative Hounsfield unit >1,000.

### DSA

Two readers (neurointerventionists #2 and #3) blinded to clinical and radiological information independently performed de-identified image interpretation through iSite PACS of cine images from fluoroscopic runs obtained on Phillips Allura biplane machine (Andover, MA) to categorize the presence or absence of CaW or atherosclerotic plaque in individual carotid arteries. Individual carotids were evaluated in a random fashion. Discrepant opinions were settled by a consensus read.

### US

Two readers (both neurosonology-credentialed and fellowship trained stroke neurologists) blinded to clinical and radiological information independently performed de-identified US image interpretation. Images were acquired with a Phillips (Andover, MA) CX50 and a L12-3 broadband linear array transducer. The studies were exported in DICOM format and supplied to the readers who reviewed images in OsiriX PACS. Carotids were evaluated in pairs/per patient. In the setting of discrepancy regarding etiological diagnosis, a consensus read was obtained. The degree of stenosis was based on peak systolic velocity (15).

### Statistical Analysis

The Shapiro-Wilk test was used to assess for normality. Continuous variables were reported as mean ± SD if normally distributed or median [IQR] if non-parametric. Categorical variables were reported as proportions. Two-sided Cohen's Kappa (κ) coefficient was used to evaluate the inter-rater agreement for the etiological diagnosis and degree of stenosis between CTA vs. DSA and CTA vs. US. Fisher r-to-z transformation was utilized to compare the agreement between patients with symptomatic and asymptomatic carotids. A *p* ≤ 0.05 was considered statistically significant. Statistical analysis was performed using IBM® SPSS® Statistics 23 (IBM®-Armonk, NY, USA) and SAS® University Edition (SAS Institute, Cary, N.C., USA).

## Results

Thirty patients/60 carotids were evaluated by CTA and 55 carotids were included (Supplementary Figure). Patients with symptomatic CaW (*n* = 20), compared to individuals with atherosclerosis (*n* = 10), were younger (49 ± 9 vs. 60 ± 8 years; *p* < 0.01), more commonly female (75% vs. 30%; *p* = 0.01), and less frequently presented vascular risk factors: Hypertension (40% vs. 100%; *p* < 0.01), hyperlipidemia (0% vs. 50%; *p* < 0.01), diabetes (10% vs. 40%; *p* = 0.05), and smoking (5% vs. 70%; *p* < 0.01), respectively.

### CTA: Inter-rater Evaluation

Fifty-five CTAs of carotids were evaluated. There was agreement in terms of diagnosis in 52/55 (94%) vessels, leading to high inter-rater correlation strength for CTA readers (κ = 0.88, *p* < 0.0001). The etiological breakdown of carotids was: CaW (*n* = 30); atherosclerosis (*n* = 15) and normal arteries (*n* = 10). Calcium deposit was noted in 14/55 (25%) vessels.

### DSA: Inter-rater Evaluation

Twenty-eight of the 55 carotids had DSA available for review by the neurointerventionists. On DSA, a median of 2.0 projections (18% of studies had ≥3.0 projections) were provided for the readers per carotid. There was agreement in 26/28 (93%) of the vessels for diagnosis. The inter-rater correlation for DSA readers was high (κ = 0.86, *p* < 0.0001).

### US: Inter-rater Evaluation

Thirty-three of the 55 carotids had US and were made available to the US readers. There was discrepant interpretation in 13/33 (39%) leading to moderate strength of agreement among readers (κ = 0.552, *p* = 0.01).

### DSA vs. CTA

When comparing the performance of DSA and CTA (*n* = 28) for etiologic definition, there was a very high strength of agreement (κ = 0.92, *p* < 0.0001) observed amongst methods. DSA readers identified correctly all normal vessels but one misdiagnosed 2 CaW as atherosclerotic lesions [CaW was seen in 43%(12/28) of the DSA group vs. 50%(14/28) of the CTA group].

### US vs. CTA

The correlation between US and CTA (*n* = 33) reads showed moderate strength of agreement (κ = 0.553, *p* = 0.001). Ultrasound diagnosed CaW less frequently [12%(4/33) vs. 42%(14/33)], and over-identified the number of normal [42%(14/33) vs. 27%(9/33)] and atherosclerotic vessels [45%(15/33) vs. 30%(10/33)] as compared to CTA, respectively. In assessing degree of stenosis generated by the CaW, CTA revealed 0% stenosis in all 14 carotids, while the US described 0% in 12 carotids, mild stenosis in 1, and low-moderate in 1.

### DSA vs. US vs. CTA:

**Figure 1:**
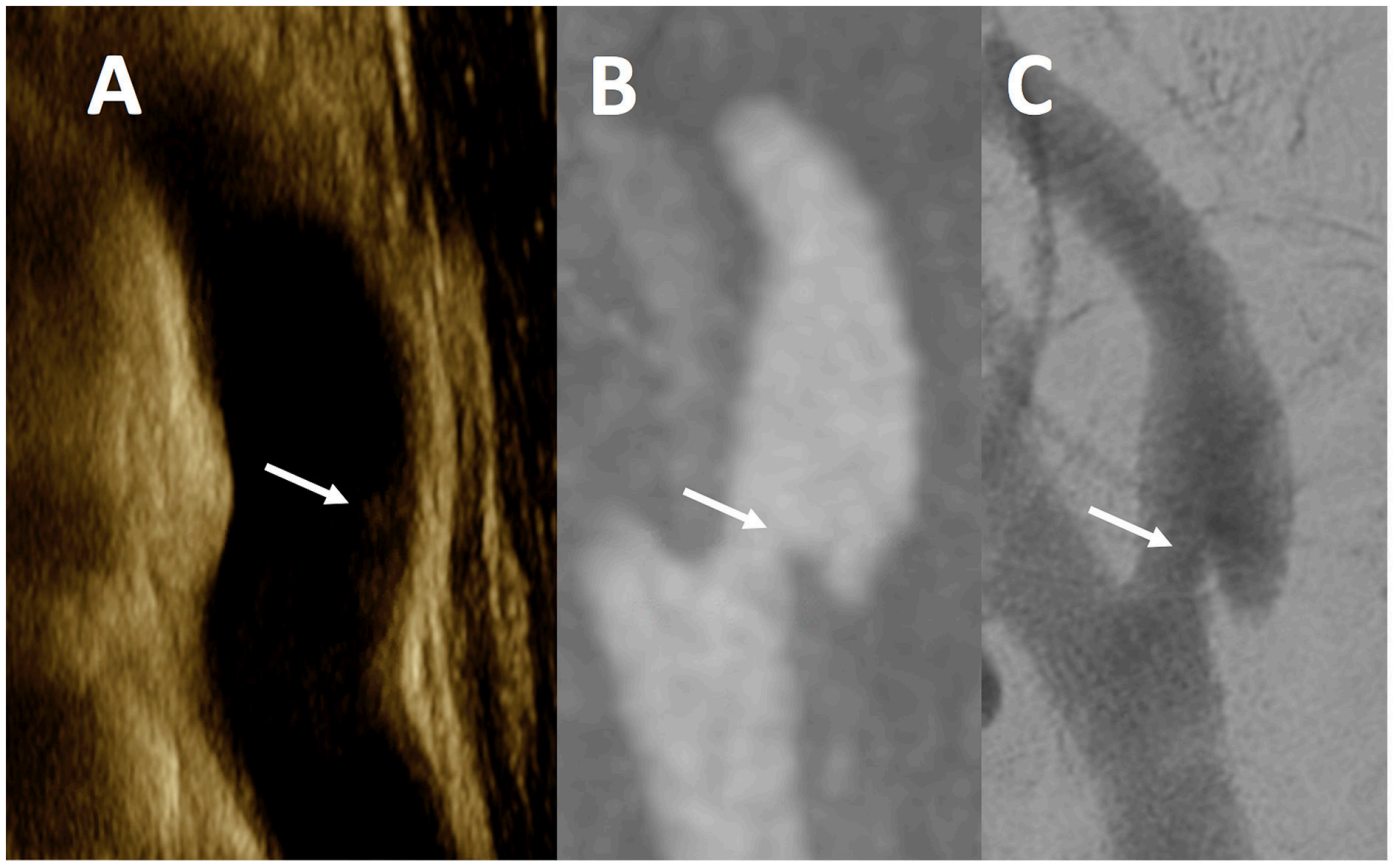
Multimodality Imaging of Carotid Web. This figure is a representative example of a CaW across all modalities in a single patient. The white arrows noted on ultrasound **(A)** as an subtle echogenic focus along the posterior wall of the internal carotid artery, and a shelf-like defect in the posterior wall of a sagittal maximum intensity projection image from CTA **(B)**, and single lateral view from DSA **(C)** of the same internal carotid artery.

In 16 vessels where all three imaging modalities were available (CTA, US, DSA), 8 carotids had CaW (Figure 1) and 8 showed atherosclerosis per CTA analysis. The correlation between DSA and CTA was perfect (κ = 1.00, *p* < 0.0001), while correlation with US and CTA was only moderate (κ = 0.62, *p* = 0.01). The strength of correlation between DSA-CTA was statistically more robust as compared to the US-CTA (Z = 3.58; *p* = 0.0003).

## Discussion

The radiographic diagnosis of CaW can be challenging and no comparative studies were available to help guiding the imaging diagnosis of these lesions. We have demonstrated that CaW can be more reliably diagnosed using DSA or CTA as compared to US.

Although prospective longitudinal data on CaW is limited, multiple prospective series have reported high rates of recurrent ipsilateral ischemic strokes in young patients with such lesions (2, 3, 16). The risk of stroke has been attributed to the pooling of blood with stagnation and consequent thrombosis in the rostral aspect of the webs (1). Although the optimal management strategy for patients with CaW has not been established, a significant proportion of patients have been reported to suffer recurrent events while on antiplatelet monotherapy (1). Therefore, early recognition and accurate diagnosis are critical since it may impact patient management and clinical outcomes. Unfortunately, the comparative accuracy of different vascular imaging methods is not known, since no studies have been reported. Therefore, it is important to understand the diagnostic performance of imaging modalities used to investigate CaW.

DSA has been described as the “gold standard” technique for clinching diagnosis of CaW (17). Although Sajedi et al. (9) advocated CTA as the technique of choice to characterize CaW and Singh et al. (3) asserted that characteristic imaging findings of CaW are best delineated on CTA, no data is available to substantiate this idea. CTA is widely available and relatively less costly (as compared to DSA and MRA). The ability to conveniently perform multiplanar reformats allows for diagnosis of even subtle webs that may only be visible on a certain directional plane. Our results confirm high inter-rater reliability of CTA to diagnose CaW (kappa = 0.88), similar to Coutinho et al. (kappa = 0.93) (8), Compagne et al. (kappa = 0.72) (11) and Sajedi et al. (kappa = 0.78) (9). A perceived limitation of single-phase CTA may be the lack of information regarding temporal flow dynamics. Time-resolved 4D CTA has been evolving and may shed light on carotid flow dynamics in the future (18). Although time-of-flight MRA is highly susceptible to flow artifact, sophisticated 3 Tesla MRI protocols are promising for lesion composition and investigation of flow abnormalities (19).

Even though the temporal and spatial resolution for DSA is higher than CTA, the risk of misdiagnosis of CaW may be increased when only two standard (frontal and lateral) projections are obtained since CaW are characteristically located on the posterior wall and the internal carotid artery has a postero-lateral orientation. In the present study, despite only two DSA views having being used in 82% of the carotids, the agreement with CTA was very high. The fact that CaW is an endoluminal projectile and smooth lesion confers DSA with high diagnostic accuracy.

The use of ultrasound to characterize CaW was first documented by Kliewer and Carroll (5) well before the advent of modern CTA technology with 3-D multiplanar reconstructions (20). The report acknowledges the limitation of ultrasound delineating between fibrotic lesions (e.g., CaW) and other echogenic intraluminal causes of vessel narrowing (atherosclerotic plaque or dissection). CaW have been described to present as inconspicuous outgrowths (16). In a series of 15 CaW diagnosed by CTA, a retrospective analysis of the US reports indicated that 40% of carotids were reported normal, while 60% were non-specifically described as hyper-echogenic lesions (21). The impression that US is less reliable for CaW diagnosis is corroborated by the present study.

The retrospective nature and use of a single institutional database may have introduced selection and sampling bias. The lack of targeted dedicated cine images through the CaW may limit lesion visualization in US, especially if the abnormality was not recognized by the sonographer during the initial image acquisition. Tilting of the probe during image acquisition may obviate the key image acquisition angle to depict the web. In this scenario, capturing a cine-image while sweeping through the carotid bulb may be useful. However, careful acquisition and interpretation of these cine-images may be time consuming. With respect to DSA, we did not include 3-D rotational angiography, nor did most carotids have more than two projections (missing oblique projections); future inclusion of this data may improve DSA modality performance.

As the clinical awareness of CaW rises, the use of non-invasive imaging modalities for diagnosis may be preferable. Based on our data, CTA may be the preferred non-invasive imaging modality over DSA and US. Given the inherent higher-spatial resolution of CTA compared to non-contrast MRA, future studies are needed to directly evaluate diagnostic accuracy of contrast MRA vs. CTA for CaW.

## Conclusion

CTA and DSA demonstrated comparable performance while US demonstrated inferior accuracy in the diagnosis of CaW.

## Data Availability

The datasets generated for this study are available on request to the corresponding author.

## Ethics Statement

This study was carried out in accordance with the recommendations of Emory University Institutional Review Board in accordance to Fibromuscular dysplasia and stroke study protocol with written informed consent from all subjects. All subjects gave written informed consent in accordance with the Declaration of Helsinki. The protocol was approved by the Emory University Institutional Review Board.

## Author Contributions

TM and DH: manuscript editing, drafting and reviewing, data analysis, and study design; RN, JG, CB, MF, and AA: manuscript editing and drafting, study reviewers.

### Conflict of Interest Statement

The authors declare that the research was conducted in the absence of any commercial or financial relationships that could be construed as a potential conflict of interest.

## Supplementary Material

Supplementary Figure Flow diagram for study design.
