# Supplementary figures and images for: Multimodality Imaging in Carotid Web
Source: Front Neurol. 2019 Mar 12;10:220. doi: 10.3389/fneur.2019.00220 (PMC6423072; doi:10.3389/fneur.2019.00220)

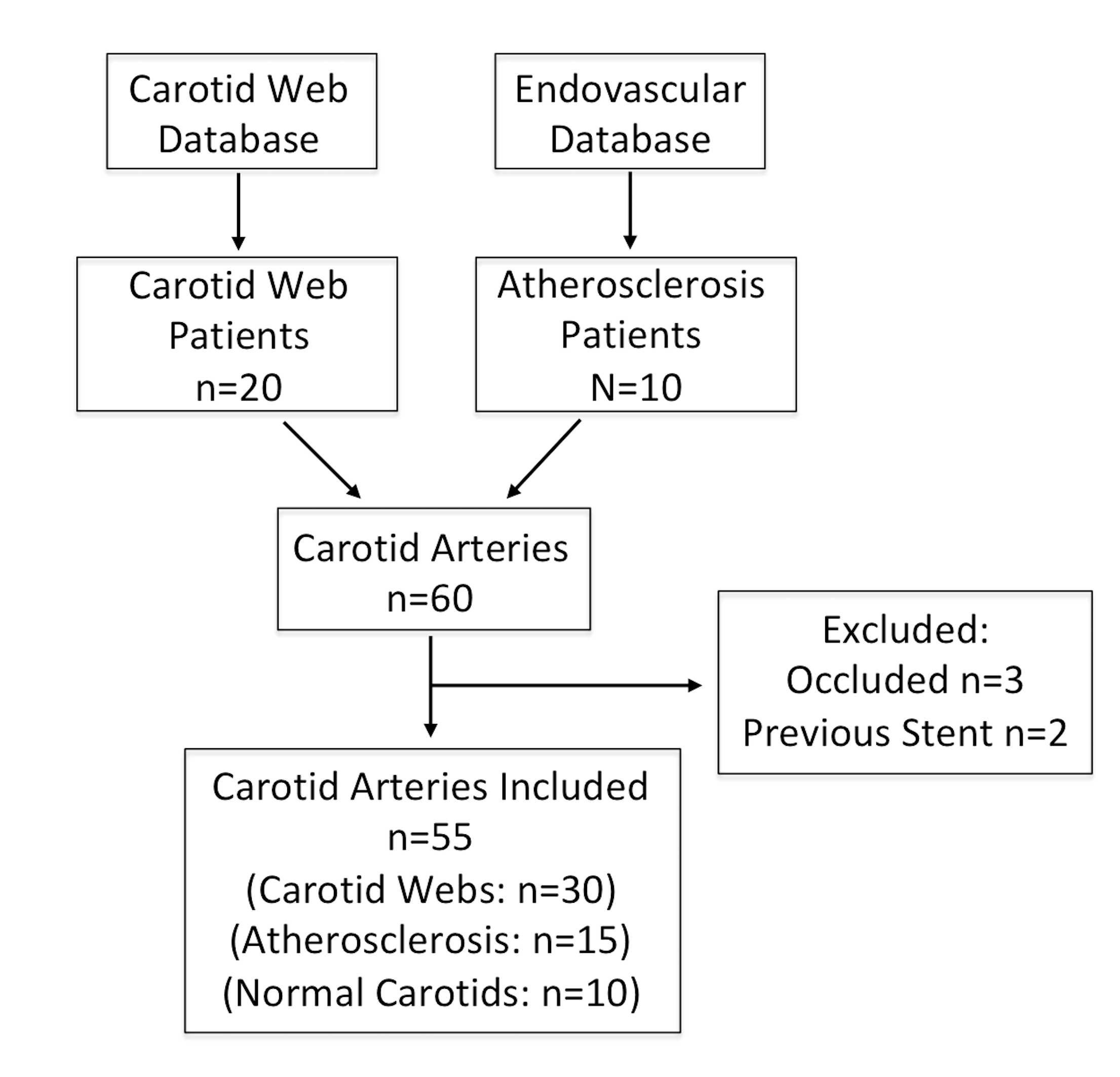

Supplement: Supplementary Figure — Flow diagram for study design. [file Image_1.TIFF]
